# A cohort study measuring SARS-CoV-2 seroconversion and serial viral testing in university students

**DOI:** 10.1186/s12879-022-07314-5

**Published:** 2022-03-31

**Authors:** Christine C. Lee, Hannah E. Segaloff, Devlin Cole, Hannah G. Rosenblum, Clint N. Morgan, Tarah Somers, Rodel Desamu-Thorpe, Monique A. Foster, Dustin Currie, Jeanne Ruff, David Payne, Thomas J. Whyte, Glen R. Abedi, John Paul Bigouette, Juliana Kahrs, Kimberly Langolf, Patrick Remington, Alana Sterkel, Patrick Kelly, Ryan P. Westergaard, Allen C. Bateman, Christopher H. Hsu, Jacqueline E. Tate, Hannah L. Kirking

**Affiliations:** 1 CDC COVID-19 Response Team, 1600 Clifton Rd. NE, Mailstop H23-6, Atlanta, GA USA; 2 Laboratory Leadership Service, CDC, Atlanta, GA 30329 USA; 3 Epidemic Intelligence Service, CDC, Atlanta, GA 30329 USA; 4 Wisconsin Department of Health Services, Madison, WI 53703 USA; 5 School of Medicine and Public Health, University of Wisconsin-Madison, Madison, WI 53705 USA; 6 University Health Services, University of Wisconsin-Madison, Madison, WI 53705 USA; 7 Agency for Toxic Substances and Disease Registry (ATSDR), CDC, Atlanta, GA 30341 USA; 8 University of Wisconsin-Oshkosh, Oshkosh, WI 54901 USA; 9 Wisconsin State Laboratory of Hygiene, Madison, WI 53718 USA

**Keywords:** SARS-CoV-2 serology, IgG antibodies, Immune protection, Antibody decline, Seroconversion

## Abstract

**Background:**

To improve understanding of the antibody response to SARS-CoV-2 infection, we examined seroprevalence, incidence of infection, and seroconversion among a cohort of young adults living on university campuses during the fall of 2020.

**Methods:**

At the beginning (semester start) and end (semester end) of an 11-week period, serum collected from 107 students was tested using the qualitative Abbott Architect SARS-CoV-2 IgG and AdviseDx SARS-CoV-2 IgG II assays. Results were matched to interim weekly surveillance viral testing and symptom data.

**Results:**

With the SARS-CoV-2 IgG assay, 15 (14.0%) students were seropositive at semester start; 29 (27.1%) students were seropositive at semester end; 10 (9.3%) were seropositive at both times. With the AdviseDx SARS-CoV-2 IgG II assay, 17 (16.3%) students were seropositive at semester start, 37 (35.6%) were seropositive at semester end, and 16 (15.3%) were seropositive at both times. Overall, 23 students (21.5%) had positive viral tests during the semester. Infection was identified by serial testing in a large majority of individuals who seroconverted using both assays. Those seropositive at semester end more frequently reported symptomatic infections (56.5%) than asymptomatic infections (30.4%).

**Conclusion:**

Differences between antibody targets were observed, with more declines in antibody index values below the threshold of positivity with the anti-nucleocapsid assay compared to the anti-spike assay. Serology testing, combined with serial viral testing, can detect seroconversions, and help understand the potential correlates of protection provided by antibodies to SARS-CoV-2.

**Supplementary Information:**

The online version contains supplementary material available at 10.1186/s12879-022-07314-5.

## Background

Severe acute respiratory syndrome coronavirus 2 (SARS-CoV-2) emerged in late 2019, resulting in millions of cases of coronavirus disease 2019 (COVID-19) nationwide. Centers for Disease Control and Prevention (CDC) developed considerations for COVID-19 prevention at institutions of higher education (IHEs), including testing upon campus move-in, SARS-CoV-2 screening testing, proper use of face masks, and significant reductions to in-person learning [1]. Despite initial efforts of IHEs in Wisconsin, the time period with the most rapid increase in SARS-CoV-2 infection rate coincided with the return of students to university campuses between September 3 and November 16, 2020 [2].

In defined populations like IHEs, antibody testing can help determine incidence of past infections and estimate relative correlates of protection from subsequent disease. Natural SARS-CoV-2 infection elicits an immune response, including generation of antibodies against viral proteins to protect from reinfection [3–5]. The degree of antibody response might correlate with symptomatic infections [6–8] and the overall severity of an individual’s illness [9, 10]. Performance characteristics from different diagnostic platforms have varying sensitivities, specificities, and positivity thresholds [11–13], while different assays evaluate various antibody isotypes (e.g., IgG, IgM, or IgA) [3, 14] or target proteins (e.g., anti-nucleocapsid versus anti-spike) [15]. Generally, antibody levels increase within the first weeks of infection, peaks after several weeks, followed by a period of decline [4, 14]. Some reports demonstrate the persistence of neutralizing antibodies for up to 5 months [16], but other reports show waning levels within several months after infection [6, 17, 18]. While antibodies are largely protective [19], a recent study among Marine recruits suggests that seropositive young adults are not guaranteed immunity against reinfection [20]. More data on antibodies as correlates of protection are needed.

We report findings from a cohort of students who had serial viral screening tests and participated in serology testing at the beginning (semester start) and end (semester end) of the 2020 fall semester at two public universities in Wisconsin. Viral testing results and reported symptoms were matched to serology results. This cohort represents a young adult population whose congregate living conditions and social behaviors pose increased risk of SARS-CoV-2 exposure and infection. The objectives of this investigation were to measure the seroprevalence of SARS-CoV-2 antibodies at two timepoints, test for seroconversion among students who had positive viral tests, evaluate trends in antibody levels among baseline seropositive students, and describe symptoms among students with confirmed SARS-CoV-2 infection.

## Methods

### Participant enrollment and serum specimen collection

College students primarily living on-campus at two large public universities in Wisconsin were invited by email to participate in free antibody testing during the fall semester of 2020. Testing was offered at central campus locations near dormitories, and a convenience sample of students were enrolled. Specimens were collected at two timepoints: semester start specimens were collected during the week following campus move-in (September 1–11, 2020) and semester end specimens were collected during the week before students returned home for the Thanksgiving holiday (November 9–14, 2020). A total of 791 and 680 serum specimens were collected at semester start and end from both universities, respectively, and a larger analysis of samples from students at University A is described elsewhere [21]. This analysis includes the subset of students who had matched serum specimens at both timepoints and had interim serial viral testing. This work was reviewed by ethical review boards at both universities, CDC, and the Wisconsin Department of Health Services, and was determined to be non-research as public health surveillance and was conducted consistent with applicable federal law and CDC policy.^1^

Following enrollment and written consent, a peripheral blood specimen was obtained. Specimens were transported in coolers containing ice packs to an on-campus laboratory where serum was separated from whole blood within two hours of collection by centrifugation. Sera were aliquoted and transported via courier to the Wisconsin State Laboratory for Hygiene (WSLH) within 72 h of collection. Sera were maintained at 4 °C from the aliquot step to the time of analysis, which was performed within seven days of collection.

At both blood collection timepoints, students were asked about history of positive viral tests from January 2020 and any symptoms at the time of first positive viral test including nasal congestion, cough, sore throat, headache, loss of smell or taste, chest pain, shortness of breath, fatigue, muscle ache, fever or chills, nausea, vomiting, or diarrhea. Symptom information captured during standard COVID-19 case investigation was confirmed from the Wisconsin Electronic Disease Surveillance System (WEDSS).

### Serologic testing

Serum specimens were initially analyzed using the qualitative Abbott Architect SARS-CoV-2 IgG assay (anti-N assay), which detects antibodies to the nucleocapsid protein of SARS-CoV-2; this assay has an index value of 1.4 as the positivity threshold (Abbott Laboratories, Abbott Park, IL) [22]. Results are reported as positive (≥ 1.4) or negative (< 1.4). Index values do not represent antibody titer levels, but are semi-quantitative values calculated from calibrator standards [22] and were used to evaluate relative changes over time. Anti-SARS-CoV-2 signal-to threshold ratios were calculated by dividing the IgG index value by 1.4 (threshold for positivity). Trends were evaluated as the percent change from semester start to semester end.

In addition, serum samples were also analyzed using the AdviseDx SARS-CoV-2 IgG II assay (anti-S assay) [23]. This assay detects antibodies to the receptor binding domain of the S1 subunit of the spike protein of SARS-CoV-2 and has a positivity threshold of  ≥ 50 arbitrary units/mL (AU/mL) of serum (Abbott Laboratories, Abbott Park, IL) [23].

### University surveillance viral testing

Regardless of the presence of symptoms, both universities requested that all students living in campus dormitories schedule bi-weekly SARS-CoV-2 viral diagnostic tests at semester start, then modified to weekly testing (beginning September 26) in response to increased positivity rates. University A had an outbreak that included on-campus housing units that coincided with student move-in, prompting increased frequency of viral testing [24]. For all students with a positive viral test result, contact tracing, isolation, and quarantine protocols were initiated, and the positive student was exempt from subsequent campus surveillance testing for 90 days based on CDC recommendations [25]. All results were reported to the local university student health system in addition to the Wisconsin Department of Public Health, with records accessible via WEDSS.

At University A, self-collected anterior nasal swabs were tested for SARS-CoV-2 using RT-PCR. RT-PCR (reverse transcription-polymerase chain reaction) testing was initially performed by Exact Sciences Laboratory (Madison, WI) using a laboratory developed test with FDA Emergency Use Authorization (EUA) [26] and was later transitioned to the UW-Madison COVID-19 Testing Laboratory at the Wisconsin Veterinary Diagnostic Laboratory, where the ThermoFisher TaqPath™ COVID-19 Combo Kit (ThermoFisher Scientific, Waltham, MA) was used [27].

At University B, anterior nasal swabs collected by university healthcare personnel were tested using the Quidel Sofia® SARS Antigen Fluorescent Immunoassay (FIA) (Quidel Corporation, San Diego, CA) [28] with confirmatory RT-PCR testing based on the university’s testing algorithm [29], which included antigen positive asymptomatic students, antigen negative symptomatic students or those with confirmed close contacts. Confirmatory RT-PCR testing was initially performed by Exact Sciences, but later transitioned to Mako Medical Laboratories using the ThermoFisher TaqPath™ COVID-19 Combo Kit [27]. If results from initial antigen test and any confirmatory RT-PCR were discrepant, the RT-PCR result was used as the definitive diagnostic result.

### Data analysis

Demographic characteristics of survey participants were analyzed using RStudio version 4.0.2 (RStudio, PBC, Boston, MA). Viral testing results obtained throughout the semester were provided by university health systems or from WEDSS, and index values from serology analyses were provided by WSLH. Semester start and semester end serology results and viral testing data were analyzed using the ggplot2 and ggalluvial packages in RStudio. Serology status, trends from semester start to semester end, and the receiver operating characteristic (ROC) curve were analyzed using GraphPad Prism v 9.0 (GraphPad Software, San Diego, CA). Welch’s unpaired T tests were used to analyze differences in seropositive students at semester start and end. Mann Whitney U tests were used to compare the number of days between positive viral test date and end of semester serology collection date. Fisher’s exact tests were used to evaluate differences in symptom reporting between symptomatic and asymptomatic students with recorded positive viral tests. p-values < 0.05 were considered significant.

## Results

### Demographics and seroprevalence

A total of 107 students (n = 70 from University A and n = 37 from University B) had serology results from both time points, symptom and prior testing data, and serial viral testing results available and comprise the study population represented in this analysis. The median age of student participants was 18 years (range of 18–22); most were female (n = 77, 72.0%), identified as White persons (n = 92, 86.0%), and non-Hispanic/Hispanic persons of other races (n = 72, 67.3%, Table 1). Most participants were freshman (n = 75, 70.1%, Table 1) and largely represented those living in on-campus housing, as most on-campus residence halls were comprised of the freshman class at both universities. These demographics were similar among participants from both universities, and data were combined for analysis.

**Table 1:** Demographics characteristics of students included in serology cohort investigation at two universities in Wisconsin

Total students: n = 107	
Characteristic	Total number (%)
Median age in years (range)	18 (18–22)
Sex	
Female	77 (72.0%)
Male	30 (28.0%)
Class	
Freshman	75 (70.1%)
Sophomore	8 (7.5%)
Junior	10 (9.3%)
Senior	11 (10.3%)
Missing/declined	3 (2.8%)
Race	
White	92 (86.0%)
Black or African American	0 (0%)
Asian	6 (5.6%)
People who identify with more than one race/ethnicity	5 (4.7%)
Missing/declined	4 (3.7%)
Ethnicity	
Hispanic or Latino	4 (3.7%)
Non-Hispanic/Hispanic persons of other races	72 (67.3%)
Missing/declined	31 (29.0%)

Among 70 student participants from University A, 7 (10.0%) were seropositive at semester start and 19 (27.1%) were seropositive at semester end (p = 0.11) by the anti-N assay (Table 2). At University B, eight of 37 (21.6%) students were seropositive at semester start and 10 of 37 (27.0%) students were seropositive at semester end (p = 0.74) (Table 2). In total, 15 (14.0%) students were seropositive at semester start, 29 (27.1%) students were seropositive at semester end (p = 0.22); 10 (9.3%) were seropositive at both time points (Table 2). These samples were also tested using the anti-S assay. Data was unavailable for two students using this assay and they were excluded from this analysis. Among 69 students from University A, 10 (14.5%) were seropositive at semester start and 29 (42.0%) were seropositive at semester end (p = 0.89) (Table 2). At University B, seven of 36 (19.4%) students were seropositive at semester start and 13 (36.1%) were seropositive at semester end (p = 0.64).

**Table 2:** Viral testing and overall serology results among students included in serology cohort investigation from two universities in Wisconsin (August 21–November 14, 2020)

Serology testing characteristics			
SARS-CoV-2 IgG assay (anti-nucleocapsid)	University A (N = 70)	University B (N = 37)	Total (N = 107)
Number of students who tested positive at semester start (%)	7 (10.0%)	8 (21.6%)	15 (14.0%)
Number of students who tested positive at semester end (%)	19 (27.1%)	10 (27.0%)	29 (27.1%)
p-value^a^	0.11	0.74	0.22

AdviseDx SARS-CoV-2 IgG II assay (anti-spike)	University A (N = 69)	University B (N = 36)	Total (N = 105)^b^
Number of students who tested positive at semester start (%)	10 (14.5%)	7 (19.4%)	17 (16.2%)
Number of Students who tested positive at semester end (%)	29 (42.0%)	13 (36.1%)	42 (40.0%)
p-value^a^	0.89	0.64	0.073

Viral testing characteristics	University A^c^ (N = 70)	University B^d^ (N = 36)^e^	Total (N = 106)
Median number of viral tests over the semester per student (range)	9 (1–13)	10 (2–15)	9 (1–15)
Number of students with positive viral test before semester (%)^f^	2 (2.9%)	2 (5.6%)	4 (3.8%)
Number of students who tested positive during serial viral testing (%)	20 (28.6%)	3 (8.3%)	23 (21.7%)

^a^Welch’s T test

^b^Data for AdviseDx SARS-CoV-2 IgG II Assay unavailable for 2 students

^c^Viral testing at University A used RT-PCR

^d^Viral testing at University B used antigen testing, with confirmatory testing by RT-PCR

^e^One student was excluded from viral testing analysis from University B due to lack of confirmatory RT-PCR results

^f^Reported positive viral tests from epidemiological surveys at semester start and end (University A) or reported through WEDSS (University B)

### Viral testing and seroconversion

Two of 70 (2.9%) students reported previous laboratory-confirmed SARS-CoV-2 infections prior to campus arrival in the survey administered at University A, and two of 36 (5.6%) students had recorded viral infections captured in WEDSS from University B prior to campus arrival (Table 2); all four were seropositive at semester start and never tested positive for SARS-CoV-2 during the semester. One student was excluded from viral testing analysis (but included in all serology analyses) because confirmatory RT-PCR information was unavailable. Two participating students from University B tested antigen positive but were RT-PCR negative during same-day follow-up testing and were considered viral test negative. Students at both universities had comparable numbers of total campus viral tests conducted between serology testing time points; a median of 9 (range 1–13) viral tests were performed per participant at University A, and a median of 10 (range 2–15) viral tests were performed per participant at University B. A total of 20 students (28.6%) from University A and 3 students (8.3%) from University B had a positive viral test during the semester (Table 2) and had fewer tests overall (average 3.4 serial tests) due to the 90-day exemption from subsequent testing following a positive test.

**Fig. 1:**
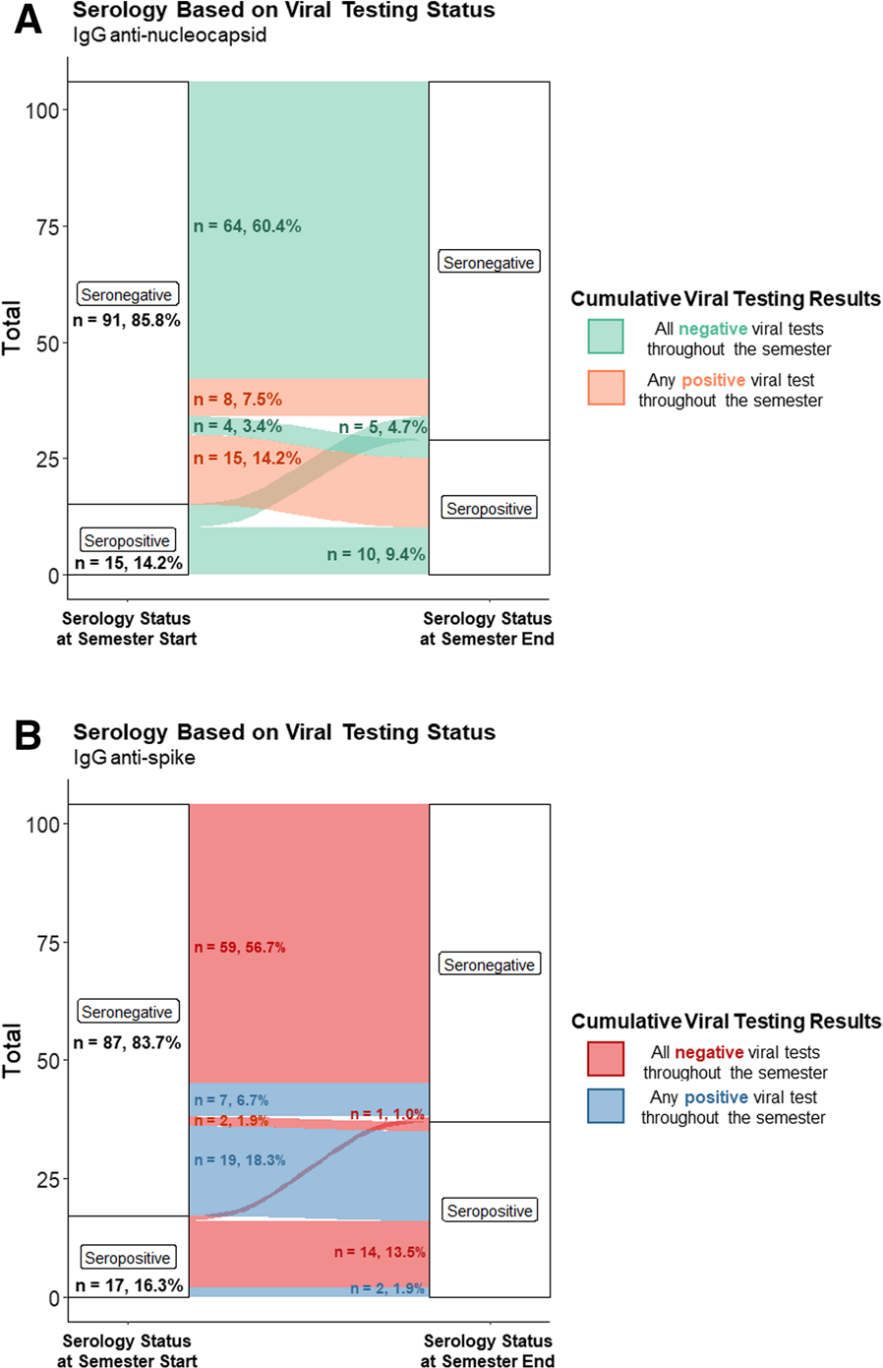
Serology status of students at the beginning and end of the semester at two universities in Wisconsin. Student serology status at the beginning of the semester (first panel) and end of the semester (third panel), and interim serial SARS-CoV-2 viral testing results (middle panel). Seroconversion in students was evaluated based on serology results at the beginning and end of the semester and from viral testing results throughout the semester. **A** Data from the anti-N assay are shown. Numbers indicate the total number of individuals who either had positive viral test (orange connecting bars) or consistently tested negative (green) during the semester. **B** Data from the anti-S assay are shown. Numbers indicate the total number of individuals who either had positive viral test (red connecting bars) or consistently tested negative (blue) during the semester

Of the 91 students who were seronegative at semester start based on the anti-N assay, 64 (60.4%) never had a positive test and remained seronegative at semester end (Fig. 1A). Four students (3.4%) did not have a recorded positive viral test, despite seroconverting from negative to positive by semester end (Fig. 1A). Three of these four students were from University B and received serial antigen tests; one student from University A only had one follow-up RT-PCR test four weeks following initial testing during campus move-in. The remaining 23 seronegative students at semester start had positive viral tests during the semester (Fig. 1A). Among these students, eight (7.5% of all participating students) remained seronegative at semester end and 15 (14.2% of all participating students) seroconverted by semester end (Fig. 1A). Overall, a total of 19 students seroconverted and 5 seroreverted by semester end.

Based on the anti-S assay, 59 (56.7%) of the total 87 seronegative students at semester start never had a positive test and remained seronegative at semester end (Fig. 1B). Two students (1.9%) did not have a recorded positive viral test but seroconverted to seropositive by semester end (Fig. 1B). The remaining 26 seronegative students at semester start had a positive viral test during the semester; seven students (6.7%) remained seronegative at semester end while 19 students (18.3%) seroconverted (Fig. 1B). Overall, a total of 21 students seroconverted and 1 seroreverted by semester end.

**Fig. 2:**
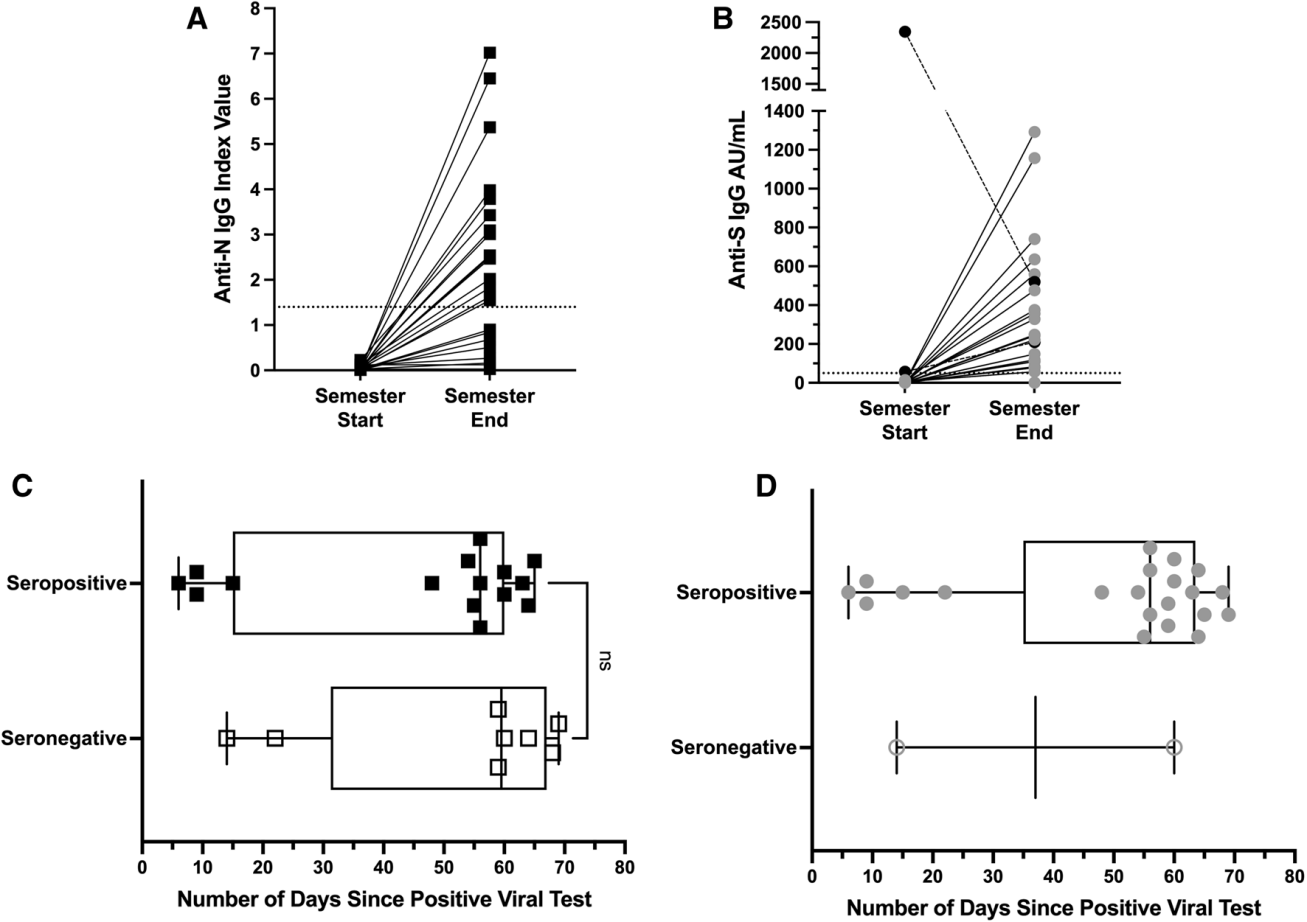
Semester start and end IgG serology for students with a confirmed positive viral test during the semester. **A** Line plots show trajectories of anti-N IgG index values from baseline to endpoint for students who had baseline negative serology and positive viral tests reported during the semester. The horizontal dotted line at index value of 1.4 indicates the threshold for positivity. **B** Number of days between positive viral test date and endpoint serology collection date, based on endpoint serology status as determined by the anti-N assay. The whiskers indicate range and boxes indicate median with interquartile range (seropositive and seronegative medians are not significant, ns, p = 0.17, Mann Whitney U Test). **C** Line plots show trajectories of anti-S IgG values (in arbitrary units/mL). The horizontal dotted line at 50 AU/mL indicates the threshold for positivity. Black dots represent two students who were seropositive at semester start but did not have confirmed positive viral tests until after serum collection. **D** Number of days between positive viral test date and endpoint serology collection date, based on endpoint serology status as determined by the anti-S assay as in **B**

Matched anti-N IgG index values from semester start to semester end for the 23 students with a positive viral test are shown in Fig. 2A. Among the 15 (65.2%) students with a positive viral test who seroconverted during the semester, the median number of days between a positive viral test and end of semester serology result was 56 days (95% CI 15–60, with a range of 6–65 days, Fig. 2B). The median number of days between a positive viral test and end of semester serology result for seronegative students was 60 days (95% CI 14–69, with a range of 14–69 days) (Fig. 2B). This difference was not statistically significant (p = 0.17).

Matched anti-spike IgG values from semester start to semester end for the same 23 students with a positive viral test are shown in Fig. 2C. Two students had positive serology at the time of blood collection (semester start) and had confirmed positive RT-PCR tests seven and eight days later; both students were from University A and did not have a previously recorded viral test (Fig. 2B). Among the remaining 19 (82.6%) students who had a positive viral test and seroconverted during the semester, the median number of days between a positive viral test and end of semester serology result was 56 days (95% CI 48–63, with a range of 6–69 days) (Fig. 2D). Two participants did not seroconvert despite having positive viral tests 14 and 60 days, respectively, before the end of semester serology.

**Fig. 3:**
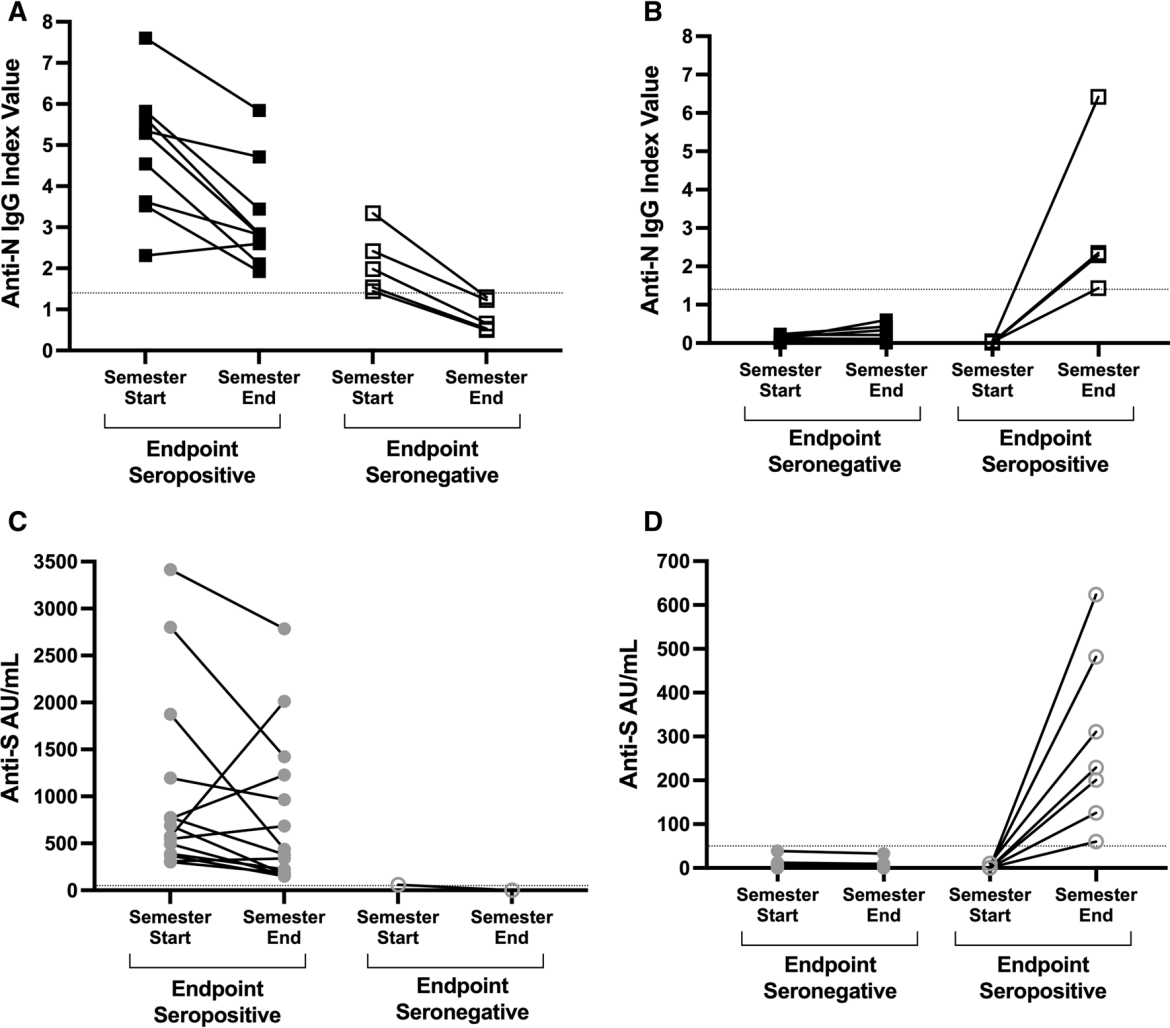
Semester start and end IgG serology index values for students with only negative viral tests during the semester. **A** Students with baseline positive serology and negative viral tests reported during the semester. Anti-N IgG index values are shown. **B** Students with negative baseline serology and negative viral tests reported during the semester. The horizontal dotted line at index value of 1.4 indicates the threshold for positivity. **C** Anti-S results from students with negative viral tests reported during the semester. **D** As in **B**, anti-S results in students with negative test results reported during the semester. Horizontal dotted line at 50 AU/mL represents the threshold of positivity

Paired IgG index values from the anti-N assay in students who never had a positive viral test are shown in Fig. 3A and B. None of the 15 students who were seropositive at semester start had a positive viral test during the semester (Fig. 3A). However, signal-to-threshold ratios calculated from IgG index values declined on average by 42% (Table 3, Fig. 3A). Five students began the semester seropositive but did not have antibodies above the positive threshold by the end of the semester (Fig. 3A). Semester start anti-N IgG index values were significantly lower for students with endpoint negative serology compared to those with endpoint positive serology (p = 0.0027). Despite the decline to seronegative results by semester end, none of these five students reported a positive viral test during the semester (i.e. no reinfection). Of the students who consistently tested viral negative throughout the semester, 64 remained seronegative and 4 seroconverted (Fig. 3B).

**Table 3:** Anti-SARS-CoV-2 signal-to-threshold ratios and percent change over time among students who were seropositive by the anti-nucleocapsid assay at semester start

Semester start IgG index value	Semester end IgG index value	Semester start signal-to-threshold ratio	Semester end signal-to-threshold ratio	Percent change (%)	Number of days between baseline and endpoint collection
7.6	5.84	5.43	4.17	− 23	63
5.82	3.44	4.16	2.46	− 41	63
5.65	2.84	4.04	2.03	− 50	69
5.34	4.71	3.81	3.36	− 12	65
5.29	2.82	3.78	2.01	− 47	64
5.29	2.83	3.78	2.02	− 47	67
4.54	2.11	3.24	1.51	− 54	68
3.62	2.82	2.59	2.01	− 22	64
3.52	1.92	2.51	1.37	− 45	64
3.34	1.3	2.39	0.93	− 61	68
2.42	1.23	1.73	0.88	− 49	70
2.31	2.6	1.65	1.86	13	68
1.98	0.66	1.41	0.47	− 67	63
1.54	0.53	1.10	0.38	− 66	68
1.44	0.5	1.03	0.36	− 65	62

IgG index values at baseline and endpoint are shown. Signal-to-threshold ratios were calculated for both baseline and endpoint IgG values based on 1.4 threshold for positivity. Percent change was evaluated for baseline and endpoint IgG index values, and the number of days between baseline and endpoint serum collection are shown

Paired anti-S IgG values from students who never had a positive viral test are shown in Fig. 3C and D. While an overall decreasing trend occurs, only one student who started the semester seropositive had endpoint negative serology (Fig. 3C). Furthermore, a total of seven students seroconverted at the end of the semester despite never testing positive by a viral test (Fig. 3D). To further examine the differences between the two serology assays, a receiver operating characteristic (ROC) analysis was performed. The anti-S assay has a higher area under the ROC and therefore has better performance in sensitivity and specificity compared to the anti-N assay (Additional file 1: Fig. 1).

### Symptomatic versus asymptomatic infections

**Fig. 4:**
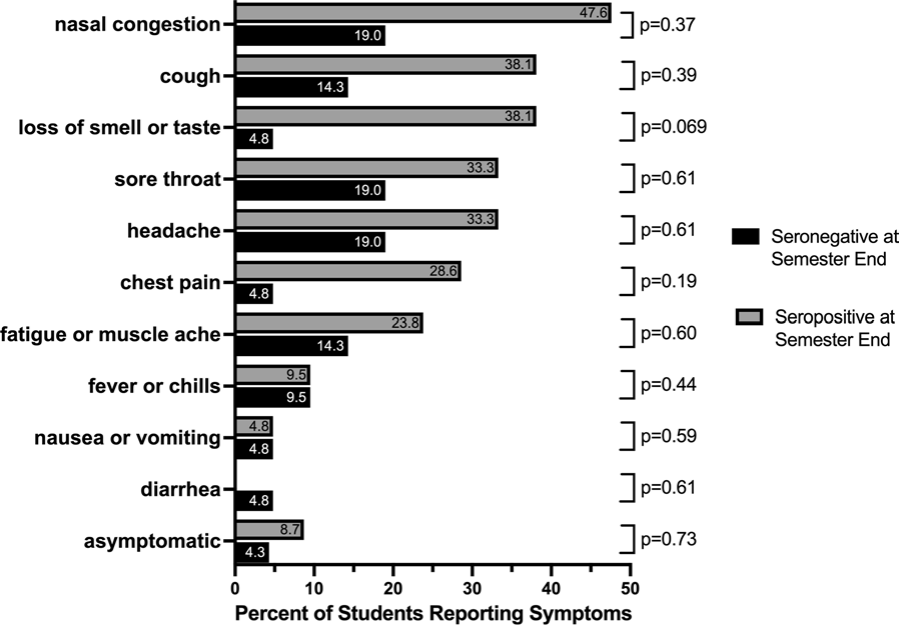
Percent of students who were seronegative and seropositive at the end of semester who reported symptoms with their SARS-CoV-2 infection. Symptoms were reported at the time of specimen collection for SARS-CoV-2 viral testing. Percentage of total reporting symptoms in students seronegative at the end of the semester (black, n = 8) and seropositive at the end of the semester (grey, n = 15) are shown in bars. Percentage of students reporting each symptom are indicated inside each bar, Fisher’s exact test was used for statistical comparisons between seropositive and seronegative students

Among the total 23 students who had positive viral tests during the semester, 20 (87.0%) reported at least one symptom. Those with positive serology by the anti-N assay (13/23, 56.5%) at the end of the semester more frequently reported one or more symptoms than students who were seronegative (7/23, 30.4%, p = 0.0093) at the end of the semester (Fig. 4). Nasal congestion, cough, and loss of smell or taste were more frequently reported by seropositive students at the end of the semester (47.6%, 38.1%, and 38.1%, respectively) than seronegative students (19.0%, 14.3%, and 4.8%, respectively; p = 0.37, 0.39, and 0.069, respectively).

## Discussion

This investigation describes SARS-CoV-2 serology testing results at two time points with interim matched serial viral tests in a cohort of relatively healthy university students. Our analysis captures the seroconversion of more than three-fourths of students with confirmed positive viral tests during the semester. Importantly, serial viral testing analysis revealed that no students who were seropositive at semester start were reinfected during the semester, suggesting that having detectable IgG antibodies could potentially be a correlate of protection from reinfection among this population during this time interval of 11 weeks. The results from this epidemiologic investigation, supported by data from widely available, routinely used, and high-throughput molecular and serological tests are important surveillance tools to help approximate seroprevalence and protection provided by antibodies.

Four students seroconverted based on the anti-N assay between semester start to semester end but did not have an interim positive viral test. Three of these four were from University B. Furthermore, seven students seroconverted based on the anti-S assay without a record of a positive viral test during the semester. Four of these seven were from University B. Potential gaps in test positivity include poor quality respiratory swabs from self-collection at University A, infections with very short periods of viral shedding missed by weekly testing, relatively infrequent serial tests due to poor adherence to requested serial testing, or false negative viral tests at University B (due to low sensitivity of viral tests in asymptomatic individuals [30]). Students who had a positive viral test but did not seroconvert may not have developed detectable antibodies [31]. Alternatively, they might have had false-positive viral test results, or detectable antibodies which rapidly fell below the threshold of positivity when tested at the end of the semester. Additional research with larger cohorts and more frequent antibody testing might provide more data on antibody duration.

We observed a decrease in signal-to-threshold ratios of anti-N IgG index values over the course of the semester in students who were seropositive at semester start, with an average percent decrease of 42%. This decline has been observed previously [18], occurs more prominently in asymptomatic or mild cases [6, 9, 10, 32], and is associated with relatively lower antibody levels immediately following infection [3]. Additional testing with the orthogonal antibody target, anti-spike protein, addressed this rapid decline as three of the four individuals who seroreverted from positive to negative by the end of the semester based on the anti-N assay remained seropositive by the anti-S assay. However, IgG index values below the threshold of positivity on a qualitative anti-N test may still be protective against reinfection, as other aspects like antibodies against other SARS-CoV-2 viral proteins like spike or the T-cell response may contribute to immunity [33, 34].

Antibody testing may be used to evaluate the overall risk of infection in local communities by determining overall seroprevalence, but there are important considerations in using this methodology. There may be differences in antibody responses among patients of different sexes, ages, comorbid medical conditions, and other characteristics [35]. Other important considerations include timing and frequency of serological testing due to antibody kinetics following infection. While informative, serology tests might underestimate the true prevalence of SARS-CoV-2 infection in a population due to the rapid decline of anti-nucleocapsid antibodies; in our study antibodies declined over an 11-week period. One option to increase assay sensitivity is to lower the threshold of positivity, allowing high “negative” values [15]—those that are just beneath the threshold of positivity but may actually be clinically protective—to be recategorized as positive. Lowering the positivity thresholds when using the Abbott Architect SARS-CoV-2 IgG assay might improve the sensitivity of the test to detect antibodies [11, 36], but will come with a tradeoff in specificity.

This investigation has several limitations. First, this study was conducted from a small sample size using convenience samples from students at two universities in Wisconsin. Participants were relatively young, healthy, and the majority identified as White females in the freshman class. Our results may not be widely generalizable to the two universities or other populations at high risk for COVID-19 such as people from some racial and ethnic minority groups [37–39] or older adults with underlying health conditions [40]. Second, our investigation was limited to serology testing at two time points, which limited our overall sample size. While anti-nucleocapsid antibody assays can be more sensitive in detecting early disease compared to anti-spike [41], this analysis captured infections that were confirmed by RT-PCR in two seropositive students by the anti-S assay sooner than the anti-N. Combination of at least two target antigens provides more insight to the immune response following SARS-CoV-2 infection [8]. Nonetheless, data from anti-nucleocapsid-based tests, including the Abbott Architect assay, provide clinically robust information [11] and is a useful tool to differentiate between natural and vaccine-induced immunity. Third, antibody neutralization studies were not performed, so definitive conclusions regarding antibody protection cannot be made. Fourth, this analysis was conducted prior to widespread Emergency Use Authorization by the FDA and availability of vaccines, and therefore does not consider protection provided through immunizations. Finally, while most students participated in viral screening testing on campus, not everyone adhered to the same frequency of testing and viral infections may have been missed due to under-sampling.

In the United States, young adults have the highest incidence of SARS-CoV-2 infections [42], and are least likely to adhere to prevention behaviors such as mask wearing, avoidance of crowds and restaurants, and physical distancing [43]. Amidst the second year of the global SARS-CoV-2 pandemic, universities continue to adjust policies for on-campus living and learning; delineating the evidence for protection by antibodies and the risk of reinfection remain important. An increasing number of convalescent individuals may have now acquired antibodies from natural infection or from vaccination. Whether or not antibodies provide correlates of protection—and the duration of that protection—will have important implications on the trajectory of the pandemic and future outbreaks and will inform the prevention strategies needed. These considerations apply both to people with a history of natural infection and to those with vaccine-induced immunity.

## Conclusions

This investigation shows the prevalence of antibodies against SARS-CoV-2 in young adults from two college campuses at the beginning and end of the semester, matched with serial viral testing results. Most students with a positive viral test seroconverted at the end of the semester. Serology testing, combined with serial viral testing, can help inform the potential correlates of protection provided by antibodies to SARS-CoV-2.

## Supplementary Information

**Additional file 1: Figure S1.** Diagnostic performance of two serology assays

## Data Availability

The datasets used generated and/or analyzed during the current study are not publicly available because this work was non-research and conducted as public health surveillance. However, summary data are available from the corresponding author on reasonable request.

## Abbreviations

CDC: Centers for Disease Control and Prevention
COVID-19: Coronavirus disease 2019
EUA: Emergency Use Authorization
FDA: Food and Drug Administration
IgA: Immunoglobulin A
IgG: Immunoglobulin G
IgM: Immunoglobulin M
IHE: Institutions of higher education
RT-PCR: Reverse transcription-polymerase chain reaction
SARS-CoV-2: Severe acute respiratory syndrome coronavirus 2
WEDDS: Wisconsin Electronic Disease Surveillance System
WSLH: Wisconsin State Laboratory for Hygiene

## Footnotes

1 See e.g., 45 C.F.R. part 46.102(l)(2), 21 C.F.R. part 56; 42 U.S.C. §241(d); 5 U.S.C. §552a; 44 U.S.C. §3501 et seq.
