# Supplementary material for: A cohort study measuring SARS-CoV-2 seroconversion and serial viral testing in university students
Source: BMC Infect Dis. 2022 Mar 31;22:314. doi: 10.1186/s12879-022-07314-5 (PMC8968700; doi:10.1186/s12879-022-07314-5)
Supplement: Supplementary file 1 — Additional file 1: Figure S1. Diagnostic performance of two serology assays [file 12879_2022_7314_MOESM1_ESM.docx]

**Supplemental Figure 1: Diagnostic performance of two serology assays**


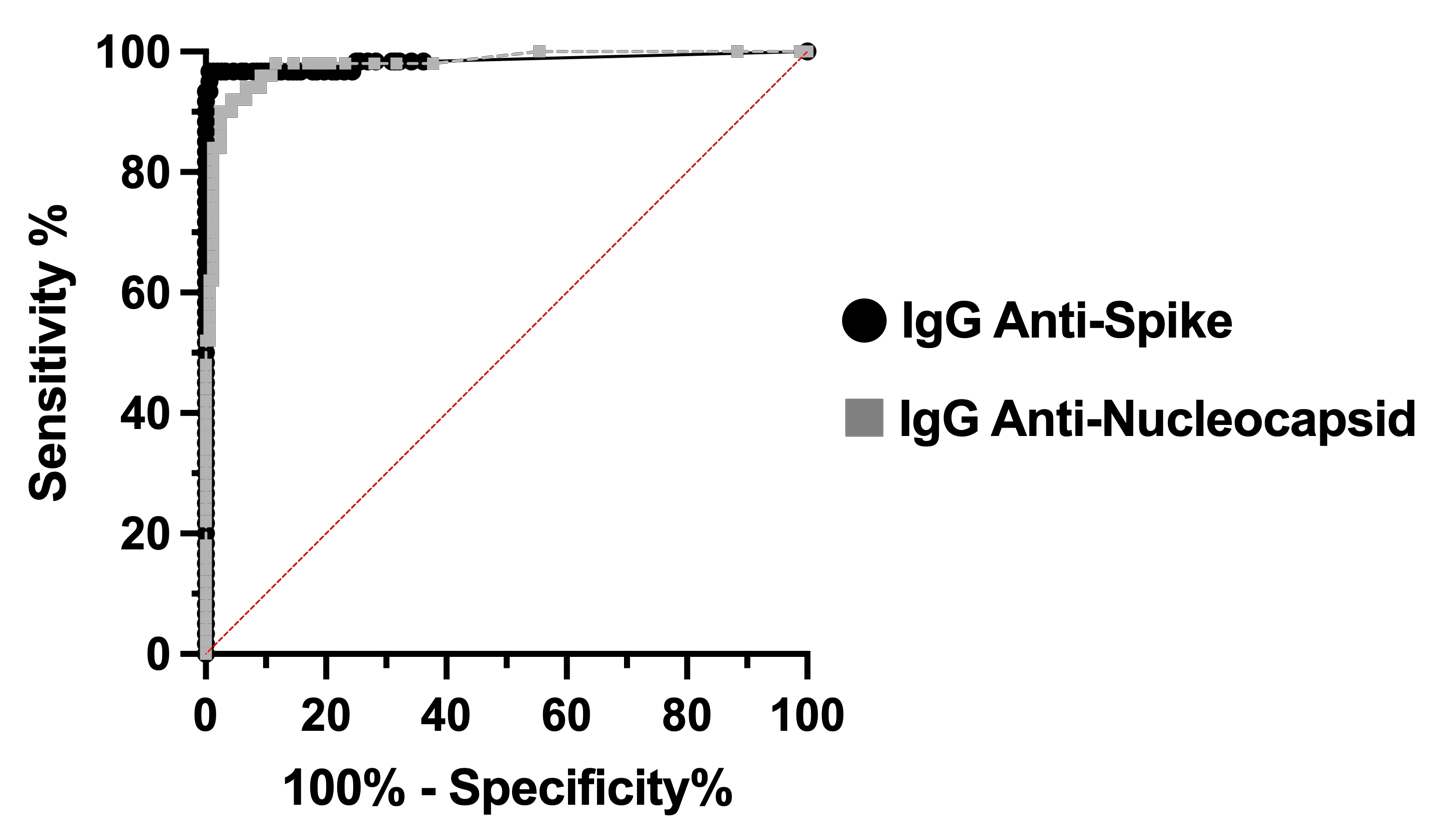


Receiver operating characteristic (ROC) curves show sensitivity and specificity of two different serology assays; one targeting immunoglobulin antibodies to nucleocapsid (IgG Anti-Nucleocapsid) and another to spike (Anti-Spike) proteins of SARS-CoV-2.
